# The Pheno- and Genotypic Characterization of Porcine *Escherichia coli* Isolates

**DOI:** 10.3390/microorganisms9081676

**Published:** 2021-08-06

**Authors:** Tanja Bernreiter-Hofer, Lukas Schwarz, Elke Müller, Adriana Cabal-Rosel, Maciej Korus, Dusan Misic, Katrin Frankenfeld, Kerstin Abraham, Olivia Grünzweil, Astrid Weiss, Andrea T. Feßler, Franz Allerberger, Stefan Schwarz, Michael P. Szostak, Werner Ruppitsch, Andrea Ladinig, Joachim Spergser, Sascha D. Braun, Stefan Monecke, Ralf Ehricht, Igor Loncaric

**Affiliations:** 1 Institute of Microbiology, University of Veterinary Medicine, 1210 Vienna, Austria; kerstin.abraham@hotmail.com (K.A.); 01245128@students.vetmeduni.ac.at (O.G.); michael.szostak@vetmeduni.ac.at (M.P.S.); joachim.spergser@vetmeduni.ac.at (J.S.); igor.loncaric@vetmeduni.ac.at (I.L.); 2 Department for Farm Animals and Veterinary Public Health, University Clinic for Swine, University of Veterinary Medicine, 1210 Vienna, Austria; lukas.schwarz@vetmeduni.ac.at (L.S.); andrea.ladinig@vetmeduni.ac.at (A.L.); 3 Leibniz Institute of Photonic Technology (IPHT), 07745 Jena, Germany; elke.mueller@leibniz-ipht.de (E.M.); sascha.braun@leibniz-ipht.de (S.D.B.); stefan.monecke@leibniz-ipht.de (S.M.); ralf.ehricht@leibniz-ipht.de (R.E.); 4 InfectoGnostics Research Campus, 07745 Jena, Germany; 5 Austrian Agency for Health and Food Safety (AGES), Institute of Medical Microbiology and Hygiene, 2340 Mödling, Austria; adriana.cabal-rosel@ages.at (A.C.-R.); franz.allerberger@ages.at (F.A.); werner.ruppitsch@ages.at (W.R.); 6 Department of Functional Food Products Development, Faculty of Biotechnology and Food Science, Wroclaw University of Environmental and Life Sciences, 51-630 Wroclaw, Poland; maciej.korus@upwr.edu.pl (M.K.); dusan@vet.bg.ac.rs (D.M.); 7 INTER-ARRAY GmbH, Forschungszentrum für Medizintechnik und Biotechnologie, 99947 Bad Langensalza, Germany; kfrankenfeld@fzmb.de; 8 BS-Immun, 1230 Vienna, Austria; office@bsimmun.at; 9 Centre for Infection Medicine, Department of Veterinary Medicine, Institute of Microbiology and Epizootics, Freie Universität Berlin, 14163 Berlin, Germany; andrea.fessler@fu-berlin.de (A.T.F.); stefan.schwarz@fu-berlin.de (S.S.); 10 Institute for Medical Microbiology and Virology, Dresden University Hospital, 01307 Dresden, Germany; 11 Institute of Physical Chemistry, Friedrich Schiller University Jena, 07745 Jena, Germany

**Keywords:** antimicrobial resistance, pig, *E. coli*, molecular characterization, microarray, colistin, WGS

## Abstract

*Escherichia* (*E.*) *coli* is the main causative pathogen of neonatal and post-weaning diarrhea and edema disease in swine production. There is a significant health concern due to an increasing number of human infections associated with food and/or environmental-borne pathogenic and multidrug-resistant *E. coli* worldwide. Monitoring the presence of pathogenic and antimicrobial-resistant *E. coli* isolates is essential for sustainable disease management in livestock and human medicine. A total of 102 *E. coli* isolates of diseased pigs were characterized by antimicrobial and biocide susceptibility testing. Antimicrobial resistance genes, including mobile colistin resistance genes, were analyzed by PCR and DNA sequencing. The quinolone resistance-determining regions of *gyrA* and *parC* in ciprofloxacin-resistant isolates were analyzed. Clonal relatedness was investigated by two-locus sequence typing (CH clonotyping). Phylotyping was performed by the Clermont multiplex PCR method. Virulence determinants were analyzed by customized DNA-based microarray technology developed in this study for fast and economic molecular multiplex typing. Thirty-five isolates were selected for whole-genome sequence-based analysis. Most isolates were resistant to ampicillin and tetracycline. Twenty-one isolates displayed an ESBL phenotype and one isolate an AmpC β-lactamase-producing phenotype. Three isolates had elevated colistin minimal inhibitory concentrations and carried the *mcr-1* gene. Thirty-seven isolates displayed a multi-drug resistance phenotype. The most predominant β-lactamase gene classes were *bla*_TEM-1_ (56%) and *bla*_CTX-M-1_ (13.71%). Mutations in QRDR were observed in 14 ciprofloxacin-resistant isolates. CH clonotyping divided all isolates into 51 CH clonotypes. The majority of isolates belonged to phylogroup A. Sixty-four isolates could be assigned to defined pathotypes wherefrom UPEC was predominant. WGS revealed that the most predominant sequence type was ST100, followed by ST10. ST131 was detected twice in our analysis. This study highlights the importance of monitoring antimicrobial resistance and virulence properties of porcine *E. coli* isolates. This can be achieved by applying reliable, fast, economic and easy to perform technologies such as DNA-based microarray typing. The presence of high-risk pathogenic multi-drug resistant zoonotic clones, as well as those that are resistant to critically important antibiotics for humans, can pose a risk to public health. Improved protocols may be developed in swine farms for preventing infections, as well as the maintenance and distribution of the causative isolates.

## 1. Introduction

*Escherichia* (*E.*) *coli* is a facultatively anaerobic Gram-negative rod with many facets. The majority of *E. coli* strains inhabit the intestinal tract of humans and warm-blooded animals as commensal bacteria in a mutually beneficial association with its hosts [1,2,3]. However, some strains of *E. coli* have acquired virulence-associated genes (VAGs), rendering them pathogenic and empowering them to play an important role as pathogens in humans and animals [3]. *E. coli* is a prominent cause for a wide range of bacterial infections in swine but might also play a role as a bacterial foodborne pathogen. In particular, VAGs enable *E. coli* to cause enteritis, urinary tract infections, peritonitis, meningitis, and septicemia in humans. In swine, *E. coli* is more prominently associated with diarrhea [4]. Depending on their VAGs, their patho-mechanisms and their clinical symptoms, *E. coli* strains are classified into numerous pathotypes. Diarrhea-associated strains include enterotoxigenic *E. coli* (ETEC), enteropathogenic *E. coli* (EPEC), enterohemorrhagic *E. coli* (EHEC), enteroaggregative *E. coli* (EAEC), and enteroinvasive *E. coli* (EIEC). Extraintestinal infections are caused by extraintestinal pathogenic *E. coli* strains (EXPEC). EXPEC are mostly innocuous gut commensals that are harmful only if they reach other body sites. They include uropathogenic strains (UPEC), or strains that are involved in septicemia in humans and animals (SEPEC), as well as *E. coli* that are involved in neonatal meningitis of humans (MENEC) [4,5,6].

*E. coli* represents a versatile and diverse enterobacterial species with a broad genetic flexibility and adaptability to constantly changing environments [7]. *E. coli* has acquired antimicrobial resistance mechanisms [8]. The genetic adaptation of *E. coli* to antibiotic exposure may select for decreased susceptibility to several antimicrobial agents [9]. Antimicrobial resistance (AMR) is recognized as a global problem in human and veterinary medicine. The high prevalence of multidrug-resistant (MDR) bacteria causes a significant concern in public health [10]. The extended use of critically important antibiotics in livestock also affects the emergence, prevalence, and dissemination of AMR [11]. VAGs and antimicrobial resistance genes are often carried on mobile genetic elements that might enable zoo-anthropogenic transfer. Therefore, monitoring the presence of pathogenic and drug-resistant *E. coli* isolates is essential for sustainable disease management in livestock and human medicine [12].

The testing and screening of virulence genes of porcine *E. coli* by single and/or multiplex PCRs is an economic factor in the frame of routine microbiological diagnostics [13]. There are numerous VAGs, but a limited number of them are usually examined by a combination of single or multiplex PCRs [13]. Accurate and time saving determination of a wide variety of genes can be accomplished using DNA microarray-based assays [14]. In the present study, we developed a microarray-based diagnostic tool combining oligonucleotides designed to detect a customized set of VAGs for use in routine diagnostics.

In Austria, there is a limited body of data describing the genomic epidemiology of *E. coli* from swine. Therefore, the objective of the present study was to characterize porcine *E. coli*, isolated during routine diagnostics, by a polyphasic approach including pheno- and genotypic susceptibility testing and whole-genome sequencing of selected isolates. For the rapid identification of virulence genes in *E. coli*, customized DNA microarray assay were developed within this study.

## 2. Materials and Methods

### 2.1. E. coli Isolates

A total of 102 *E. coli* isolates of suckling and weaning pigs were included in the present study. All isolates were gut-associated and were obtained during routine bacteriological diagnostics at the Institute of Microbiology of the University of Veterinary Medicine Vienna, Austria and from BS-Immun GmbH Vienna, Austria. All isolates originated from clinical samples received from third parties and therefore were not subject to reporting obligations of the Ethics and Animal Welfare Commission of the University of Veterinary Medicine Vienna. Isolates were stored at −80 °C until further examination.

### 2.2. Antimicrobial Susceptibility Testing

Antimicrobial susceptibility testing was performed by agar disk diffusion according to the CLSI [15]. *Escherichia coli* ATCC^®^ 25,922 served as the quality control strain. The following antimicrobials were used: ampicillin (10 µg), piperacillin (10 µg), cefotaxime (30 µg), ceftazidime (30 µg), cefepime (30 µg), aztreonam (30 µg), meropenem (10 µg), imipenem (10 µg), gentamicin (10 µg), amikacin (30 µg), tobramycin (10 µg), ciprofloxacin (5 µg), trimethoprim–sulfamethoxazole (1.25/23.75 µg), tetracycline (30 µg), fosfomycin (200 µg), and chloramphenicol (30 µg) (Becton Dickinson, Heidelberg, Germany). Isolates were further examined for extended-spectrum β-lactamase (ESBL) production by combination disk tests using cefotaxime and ceftazidime with and without clavulanic acid (Becton Dickinson, Heidelberg, Germany) [15]. Furthermore, cefoxitin (30 μg) (BD, Heidelberg, Germany) was utilized to detect AmpC β-lactamase-producing (AmpC) phenotypes. Minimal inhibitory concentration of isolates mobile colistin resistance (*mcr*) determinants were screened by broth microdilution testing method in accordance with the CLSI document VET01-A4 [16]. Colistin susceptibility testing was interpreted according to the CLSI document MR01 [17]. Escherichia coli ATCC^®^ 25,922 served as quality control strain. *E. coli* isolates displaying the AmpC phenotype were analyzed for mutations in the chromosomal *ampC* promoter/attenuator region as described previously [18]. The following resistance genes were screened via PCRs: *bla*_CMY_, *bla*_CTX_, *bla*_OXA-1_, *bla*_OXA-2_, *bla*_SHV_, *bla*_TEM_, *sul1*, *sul2*, *sul3*, *dfrA1*, *dfrA12*, *dfrA14*, *dfrA17*, *dfrA19*, *strA*, *strB*, *aadA1*, *aadA2*, *aadA4*, *aadA5*, *aadB*, *qepA*, *qnrA*, *qnrB*, *qnrC*, *qnrD*, *qnrS*, *aac(6′)-Ib-cr*, *catA1*, *cfr*, *cmlA1*, *floR*, *tet*(A), *tet*(B), *tet*(C), *tet*(D), *tet*(E), *tet*(G) as described elsewhere [19,20]. In addition, the genes *bla*_CMY_, *bla*_CTX-M_, *bla*_SHV_, and *bla*_TEM_ were sequenced after PCR amplification. All amplicons in the present study were sequenced at LGC Genomics, Berlin, Germany. Sequences were aligned with BLAST (Basic Local Alignment Search Tool. Available online: https://blast.ncbi.nlm.nih.gov/Blast.cgi, accessed on 29 July 2021) and compared with reference sequences available in GenBank and the National Center for Biotechnology Information (NCBI) database (Beta Lactamase Data Resources. Available online: http://www.ncbi.nlm.nih.gov/pathogens/beta-lactamase-data-resources/, accessed on 29 July 2021). PCR for plasmid-mediated colistin resistance genes, *mcr-1*, *mcr-2*, *mcr-3*, *mcr-4*, *mcr-5*, was performed according to the protocol of European Union Reference Laboratory for Antimicrobial Resistance [21]. The quinolone resistance-determining regions (QRDR) of *gyrA* and *parC* in ciprofloxacin-resistant isolates were amplified by PCR and sequenced [22].

### 2.3. Bicocide Susceptibility Testing

Biocide susceptibility testing was performed according to the previously established protocol by Schug et al. [23]. Established minimal inhibitory concentration (MIC) values of investigated biocides on reference strains are shown in Supplementary Materials Table S2. Benzalkonium chloride (Acros Organics, Geel, Belgium, 21541), as a representative of the quaternary ammonium compounds, was tested at concentration ranges 0.000015–0.016%; chlorhexidine (Sigma-Aldrich, Schnelldorf, Germany, 55-56-1), as a representative of cationic compounds, was tested at concentration ranges 0.000015–0.002%; glutardialdehyde (Chempur, Piekary Slaskie, Poland, 424610240), as a representative of aldehydes, was tested at concentration ranges 0.0075–1%; and isopropanol (99.9%, PHPU Eurochem BGD, Tarnow, Poland), as a representative of alcohols, was tested at concentration ranges 1–14%. The method was performed in 96-well polystyrene microtiter plates with U bottom (Sarstedt, Numbrecht, Germany, 82.1582.001). The bacterial inoculum was prepared according to the CLSI standard (Clinical and Laboratory Standards Institute, 2020), using Trypticasein soy broth (BioMaxima, Lublin, Poland, PS 23-500). The final concentration of bacteria inoculated into the wells was 2.5–5 × 10^5^ CFU/mL.

### 2.4. Clonal Relatedness of E. coli and Whole-Genome Sequencing

*E. coli* DNA was extracted as previously described [24]. Isolates were phylotyped using the quadruplex assignment method [25]. Clonal relatedness of *E. coli* isolates was assessed by two-locus sequence typing, or “CH-clonotyping”, using combined data of *fumC* and *fimH* sequences as described by Weissman et al. [26]. Allele and CH clonotype numbers were used for goeBURST analysis using PHYLOViZ [27]. Thirty-five selected *E. coli* isolates were analyzed by whole-genome sequencing (WGS), which was performed by isolating bacterial DNA using the MagAttract HMW DNA Kit (Qiagen, Hilden, Germany). Ready-to-sequence libraries were prepared using Nextera XT DNA Library Preparation Kit (Illumina, San Diego, United States). Sequencing was performed on the Illumina MiSeq platform [28]. De novo assembly of the 300 bp paired-end reads was conducted using SPAdes 3.9.0 [29]. WGS data analysis was performed with SeqSphere+ software (Ridom, Münster, Germany). To assess the genetic relatedness between the *E. coli* isolates, multi-locus sequence typing (MLST) and core genome multi-locus sequence-based typing (cgMLST) were performed as previously described [30]. To identify acquired resistance genes or chromosomal mutations, Comprehensive Antibiotic Resistance Database [31] as well as ResFinder 4.1 [32,33] were used. Genes associated with biocide resistance were compared with BacMet database (Antibacterial Biocide and Metal Resistance Genes Database. Available online: http://bacmet.biomedicine.gu.se/, accessed on 29 July 2021) [34]. Virulence genes were identified using VirulenceFinder [35,36]. CH types were characterized as mentioned above. Serogenotypes were analyzed by SerotypeFinder [37]. *E. coli* phylotypes were extracted from WGS by Clermont typing [38]. The presence of plasmids was determined using PlasmidFinder [39]. Probability prediction of the location of a given virulence or antibiotic resistance gene was achieved by applying mlplasmids trained on *E. coli* [40]. Posterior plasmid probability (ppp) scores ≥0.7 at a minimum contig length of 700 bp indicate that a given contig sequence is plasmid-derived. For selected contigs with lower ppp scores, BLAST analyses against the *Enterobacterales* nucleotide collection at NCBI were performed. Plasmid probability was assumed for mlplasmid scores > 0.699 or if BLAST analyses identified *E. coli* plasmids for at least 90% of contig length with >90% identity. The genomes of WGS isolates were deposited under PRJNA728557 in the NCBI BioProject database.

### 2.5. Microarray-Based Detection of Virulence-Associated Genes

A set of virulence genes was determined for all isolates using a DNA microarray-based technology developed in the present study frame. The technology is based on methods as described previously [41], and custom-made microarrays from INTER-ARRAY (INTER-ARRAY by fzmb GmbH, Bad Langensalza, Germany) were used according to manufacturer’s instructions. The complete list of virulence-associated genes can be found at INTER-ARRAY website (Virulence Genes for Manuscript. Available online: https://www.inter-array.com/porcineEcoli/VirulenceGenesformanuscript_supplementary_material.xlsx, accessed on 29 July 2021). A split network tree was used to visualize similarities between hybridization patterns as described previously [13].

## 3. Results

### 3.1. Antimicrobial Susceptibility Testing

All isolates were susceptible to amikacin and carbapenems. Out of the 102 *E. coli* strains, 79.41% were resistant to at least one of the remaining antimicrobial agents tested. Twenty-one isolates displayed an extended-spectrum β-lactamase (ESBL) phenotype, whereas a single isolate displayed an AmpC phenotype. In total, 36.27% of the isolates exhibited an MDR phenotype [10]. The majority of isolates were resistant to ampicillin (61.75%) and/or tetracycline (58.81%). Further resistance rates were found to piperacillin (26.46%), sulfamethoxazole–trimethoprim (23.53%), cefotaxime (13.71%), chloramphenicol (11.75%), ceftazidime (8.81%), cefepime (7.83%), gentamicin (6.85%), fluoroquinolone (5.87%), aztreonam (4.90%), tobramycin (3.91%), and fosfomycin (1.95%). A total of 2.94% of all investigated isolates exhibited elevated colistin MICs of ≥4 µg/mL. All results of antimicrobial susceptibility testing are summarized in Table 1 and Table 2.

### 3.2. Characterization of Genotypic Antibiotic Resistance

In 13.71% of the isolates, genes from the *bla*_CTX_ family were detected alone or combined with other *bla* genes. One of the isolates displayed an AmpC phenotype and carried a *bla*_CMY-2_ gene. The most prevalent β-lactamase genes detected were *bla*_TEM-1_ (56.00%) followed by *bla*_CTX M-1_ (13.71%). Three isolates carried the mobile colistin resistance gene *mcr-1.1*.

The *gyrA* and *parC* sequences of 13.72% ciprofloxacin-resistant isolates were analyzed and revealed mutations that resulted in the following amino acid substitutions: 10.78% of the isolates had a Ser83Leu, one isolate a Ser83Ala, and another 10.78% of isolates an Asp87Asn substitution in *gyrA*, while in *parC* 11 isolates displayed a Ser80Ile, 1.96% of the isolates showed Glu84Gly mutation while one isolate revealed a Cys56Thr substitution. A total of 1.96% of all isolates had an Ile355Thr mutation in *parE*. Results are listed in Table 1.

### 3.3. Biocide Susceptibility Testing

The obtained MIC values of all tested biocides against ATCC strains, including *E. coli* ATCC 10,536, were in the acceptable susceptibility. MIC values of benzalkonium chloride (BAC) for all clinical *E. coli* isolates ranged from 0.0005% to 0.002%. The obtained BAC MIC values were 0.0005% for 1.9% of isolates (2/104), 0.001% for 54.7% (59/104) of isolates, and 0.002% for 41.3% (43/104) of isolates. Chlorhexidine (CHX) MIC values comprised seven dilutions steps from 0.00003% to 0.002%. In comparison to BAC with a unimodal distribution, a bimodal MIC distribution was seen for CHX. This bimodal distribution might point towards a possibly acquired resistance property for the isolates with CHX MICs of 0.00025%. For glutaraldehyde (GLU), unimodal MIC distribution comprising five dilution steps (0.03% to 0.5%) was observed. Except for one isolate with an MIC of <1%, the remaining isolates had isopropanol (ISO) MICs from 2% to 10%. The results of the biocide susceptibility testing of *E. coli* are shown in Supplementary Materials Tables S1 and S2.

### 3.4. E. coli Phylotyping

Among all *E. coli* isolates, the most dominant phylogenetic group was A (50.98%), followed by B1 (25.48%), while the remaining belonged to C (8.81%), D (5.87%), B2 (3.91%), F (1.95%), E, G, and clade 1 (each 0.97%). Results of *E. coli* phylotyping are shown in Table 1 and Table 2 and Supplementary Materials Table S3.

### 3.5. E. coli Clonotyping

**Figure 1:**
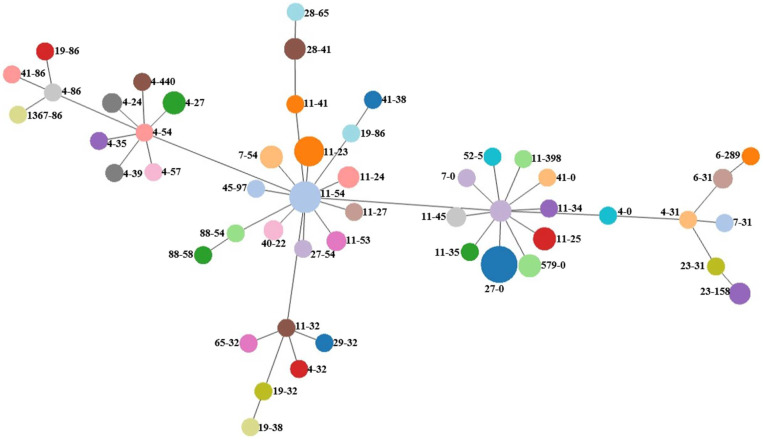
goeBURST diagram for the CH clonotyping dataset of *E. coli* isolates. An eBURST diagram was calculated using PHYLOViZ with the goeBURST algorithm. *E. coli* isolates were grouped according to their CH profiles.

The *fumC* and *fimH* (CH) typing divided all isolates into 51 distinct CH clonotypes and revealed the clonal relatedness of 12 isolates (CH27-0), 9 isolates (CH11-54) and 8 isolates (CH11-23). *E. coli*-predicted CH clonotype CH40-24 was clearly determined in isolates 24_8 and 99_74. The relatedness of isolates is visualized in Figure 1.

### 3.6. Whole-Genome Sequencing (WGS) of Selected E. coli Isolates

In our study, 35 isolates were analyzed by whole-genome sequencing (WGS). WGS revealed a total of 16 distinct STs. The most common sequence type was ST10 (*n* = 6), which clustered together by cgMLST. Further sequence types were ST100 (*n* = 5), ST354, ST131 (*n* = 2 each), and singletons ST6404, ST6365, ST1112, ST1079, ST760, ST744, ST641, ST117, ST101, ST56, ST42, and ST23. New sequence types could be obtained in three isolates: ST12008 (37_21), ST12009 (46_30), ST12010 (98_73).

The WGS analysis revealed 12 different serogenotypes (WGS-predicted serotypes). The remaining 23 strains were O-non-typeable with 11 different H types. Three isolates could not be assigned to a known serotype. Isolates belonging to O25:H4 were detected in two cases. Three isolates carrying the gene *stx2e* could be assigned to serotype O138:H14. Another *stx2*-carrying isolate belonged to serotype O121:H10.

In total, 6 out of 35 Shiga toxin-producing *E. coli* were detected in the present study carrying the genes *stx2*, *stx2e*, *stx2A* and *stx2B*.

Two out of 35 isolates belonged to the successful evolutionary line ST131 and could be assigned to phylogroup B2 (*fimH*22). Isolate 24_8 was a *bla*_TEM-1C_ and *bla*_CTX-M-1_-producing ESBL *E. coli* whereas isolate 99_74 produced only *bla*_TEM-1C_. Both strains revealed mutations in the QRDR of *gyrA* and *parC* and were multidrug-resistant. Virulence potential for both strains was inferred by the detection of multiple VAGs determining the UPEC pathotype. Virulence profile similarity among the two isolates was high and the types of virulence genes presented in these strains were coding for adhesins, toxins, siderophores, hemolysins, and protectins.

PlasmidFinder was used for the analysis of WGS data and revealed the presence of the plasmid replicons IncFIB(AP001918), IncFIC(FII), IncHI2, IncHI2A, IncX1, IncFII, IncN, IncY, IncI1-I(Alpha), IncFIA, IncQ1, p0111, IncFII(pHN7A8), Col(MG828), IncR, IncFIB(H89-PhagePlasmid), IncFII(29), IncFII(pCoo), Col156, IncFII(pSE11), IncI2(Delta), IncI2, IncFII(pRSB107), Col440II, ColpVC, IncX4 and IncB/O/K/Z. IncX4 was identified as the replicon of all *mcr-1*-carrying plasmids. IncFIB(AP001918) plasmids were predominant (27 of 35) and carried the VAGs *ompT*, *hlyF*, *cia*, and *etsC*, followed by IncI1-I(Alpha) plasmids carrying *cia* and *bla*_CTX-M-1_ and the IncX1 plasmids carrying *bla*_TEM-1B_. IncFII and IncFII(pCoo) carried *traT*. The full list of VAG and AMR genes and their predicted plasmid probability are shown in Table 1, Supplementary Materials Tables S3–S6.

### 3.7. E. coli Pathotyping

All isolates carrying VAGs and VAGs related to pathogenic *E. coli* subtypes were frequently detected. A total of 30 genes were screened by using microarray-based diagnostics. The adhesion gene *fimH* was present in all but one isolate and therefore was the most frequent gene of the adhesins category. The iron acquisition gene *iucD* was found in 24 isolates and was always represented together with the fimbrial gene *papC*. Among toxin-encoding genes, *astA* was the most predominant (*n* = 27) gene, followed by *itcA* (*n* = 13). The shigatoxin *stx2e* gene was detected in five isolates and the gene *hlyA* (*n* = 9) occurred more often than the *cnf1* gene (*n* = 3). WGS detected the toxin-associated gene *sta1* in four isolates. Of all analyzed isolates, the combination of the VAGs *fimH*, *papC* and *iucD* characterizing the UPEC pathotype was the most frequent one (23.52%), followed by the combination of a fimbrial gene/adhesion gene and a toxin gene characterizing the ETEC pathotype (22.54%). Further pathotypes were EDEC (4.90%), atypical ETEC and EPEC (each 3.92%), STEC (0.98%) and UPEC with enterotoxin (2.94%). In total, 40.19% of all *E. coli* isolates could not be assigned to a specific pathotype.

## 4. Discussion

This study aimed to characterize *E. coli* isolates from pig farms in Austria by using pheno- and genotyping methods as well as WGS. Resistance to antimicrobial agents was found in 81 (79%) isolates and 37 isolates met the MDR definition of Sweeney and colleagues [10]. Twenty-one isolates were susceptible to all antimicrobial agents tested. Resistance rates to penicillins (61.73%) and tetracyclines (58.81%) were similar to results of previous studies where penicillins and tetracyclines were the most common antibiotics with AMR in global pig production [42]. The distribution of resistance rates is similar to that in other European studies. Especially, an increased resistance to ampicillin was already reported in the EFSA surveillance program [43]. The variation in resistance in pathogenic *E. coli* was broad. This emphasizes the importance of performing antimicrobial susceptibility testing after pathotype identification for determining prognosis and guiding clinical management [44].

Colistin is considered by the WHO as a last-resort agent in the treatment of severe bacterial infections caused by multi-drug resistant Gram-negative bacteria [11]. Different genetic mechanisms are known to lead to colistin resistance. In particular, for isolates showing reduced susceptibility to colistin, this may be conferred by chromosomal alterations in *pmrAB* genes, which encode a two-component signal transduction system regulating the endogenous LPS modification system [45,46,47]. In 2015, the emergence and also the spread of mobile colistin resistance (*mcr*) genes were detected [48]. Although only three isolates in this study carried a MCR resistance gene, namely *mcr-1.1*, there is a scarcity of surveillance studies focusing on MCR genes in both human and veterinary medicine in Austria. Indeed, Austrian surveillance programs until now have not mentioned the presence of any colistin-resistant *E. coli* [49]. Only single reports from human medicine [50] and a study on the Austrian pig population reported the presence of MCR genes [50] previously. Regarding co-resistance, the fact that two of the *mcr-1.1*-positive isolates showed MDR to penicillins, tetracyclines and trimethoprim–sulfamethoxazole highlights the threat of these clones to therapeutic choices [45]. In animal production, colistin is extensively used for metaphylactic and therapeutic purposes, which may contribute to increasing levels of colistin resistance [45]. For this reason, the European Medicine Agency has raised serious concerns in regard to the use of colistin in animals and the increasing risk for humans that this antimicrobial resistance poses [43].

In addition to colistin, fluoroquinolones are critically important antimicrobials and sometimes they are the sole or one of limited available therapies to treat serious bacterial infections in people (EARS Net Reports. Available online: https://www.ecdc.europa.eu, accessed on 29 July 2021). Resistance to fluoroquinolones among the investigated *E. coli* isolates was observed in 14/102 isolates (13.7%). Although results must be compared with caution because of the different methodologies performed, the proportion of samples with resistance to fluoroquinolones was lower than in other studies performed on humans, which was revealed to be 18.2% on average [51].

Different *E. coli* lineages are responsible for animal as well as for human *E. coli* infections, with previous studies having identified food and food animal reservoirs as sources for zoo-anthropogenic *E. coli* clones [52]. A study conducted on ESBL-positive *E. coli* isolates of human and animal origin in the Netherlands, the UK and Germany revealed that human *E. coli* isolates in the three countries were more closely related to one another than to isolates from animals [53]. In our study, we found isolates of distinct *E. coli* clonal lineages, including the specific international high-risk clone O25:H4-ST131-H22, which emphasizes its wide distribution and would be the first report of ST131 in pigs of Austrian origin. In addition, recent studies demonstrated the potential of *E. coli* O25:H4-ST131 to serve as a foodborne UPEC [54] and revealed the close relationship of human and porcine ST131 strains [55]. Indeed, enhanced virulence and antimicrobial resistance were compared with other *E. coli* ST131 strains from our recent work [56]. Interestingly, a number of virulence genes, encoding colonization, iron uptake, and biofilm formation, which are key enabling factors for the clinical success of ST131 [54,56,57,58], were present in both isolate types (24 VAGs in 24_8, 26 VAGs in 99_74).

Concerning *E. coli* ST10, an ancestral and ubiquitously occurring lineage comprising both commensal and pathogenic strains, it was detected in six out of 35 sequenced isolates. All but one isolate showed MDR, including a plasmid-predicted carriage of the *mcr-1.1* gene (IncX4) in two isolates and *bla*_CTX-M-1_ (IncI1-I(Alpha)) in one isolate. Previous studies confirmed ST10 as the dominant ST from swine in Northern Europe with a broad host range and association with hospital- and community-acquired infections [59]. Shepard et al. [60] found that ST10 is one of the main *E. coli* clonal complexes associated with porcine ETEC, and Garcia et al. identified ST10 as primarily responsible for *mcr-4* spread [61]. Nevertheless, more investigations are necessary to verify if *E. coli* from porcine sources may be derived from the same bacterial lineages or share common evolutionary roots with human isolates.

The reporting of STEC O26 infections has been steadily increasing in the EU due to improved diagnostics of non-O157 sero-pathotypes (EARS Net Reports. Available online: https://www.ecdc.europa.eu, accessed on 29 July 2021). Among characterized *E. coli* strains, an atypical enteropathogenic *E. coli* (aEPEC), O26:H11_ST88, was detected. Besides the intimin (*eae*), which confers the ability to cause attaching and effacing (AE) lesions, the strain harbored heat-stable toxin gene *astA* and a further 20 VAGs. Previous studies described aEPEC as a possible progenitor of *stx*-producing O26:H11 STEC that is a major pathogen by causing severe gastrointestinal infections in animals and humans [62] and hemolytic–uremic syndrome (HUS) in humans [63]. Further studies indicated that aEPEC isolates may be able to acquire *stx* by integrating the s*tx*-prophage into their genome and further function as STEC [64]. In addition, the isolate in our study was MDR and harbored a plasmid-predicted *mcr-1.1* gene.

ETEC strains are recognized as the most common cause of porcine neonatal diarrhea (ND) and PWD in pigs [44], and were found in 23 of the investigated isolates. Interestingly, the pathotype UPEC was found to be the most common (24 isolates), although collected samples were mainly associated with ND and PWD. In total, 41 isolates could not be assigned to a specific pathotype because of lacking a specific combination of VAGs, or because of harboring VAGs that are specific for more than one pathotype. This circumstance may confirm expectations of Robins-Browne et al. and Müller et al. [3,65] that some of the typing schemes in current use will eventually be replaced, allowing more pathotypes to be identified (2016).

Phylogenetic analyses found groups A and B1 to be the most common, which corresponded to the results of similar studies [66]. Phylogroup B2 was represented by 4/102 isolates, all of which represented the UPEC pathotype, including both ST131 isolates, as previously confirmed by Nicolas-Chanoine et al. [67].

The plasmid types IncF, IncI and IncX, carrying VAGs and AMR genes, were found. These findings are a cause for concern, as these elements can easily be transferred from animal host pathogens to human pathogens, increasing their AMR and virulence [8]. lncF is the most frequently described plasmid type found in *E. coli* of human and animal sources. Interestingly, our investigation revealed that the *traT* gene, which codes for surface exclusion, was IncFI1-associated [68]. In a single isolate, *bla*_CTX-M-1_ was predicted to be on an lncl1 plasmid. Such plasmids are predominantly described as *bla*_CTX-M-1_ carriers in *E. coli* of European poultry and are further considered as a possible source for human infections [69]. In our study, three of 102 isolates carried the *mcr-1.1* gene on an IncX4 plasmid, which is in agreement with other works on Salmonella and *E. coli* isolates obtained from human and animal sources where IncX plasmids are also shown to carry *mcr* genes [69].

Biocides are applied as an integral part of infection control in pig production and slaughterhouses. The selection of bacteria with reduced susceptibility to disinfectants has already been confirmed [70]. In our study, we investigated biocide susceptibility and revealed unimodal MIC distributions for benzalkonium chloride, glutardialdehyde and isopropanol. In comparison, a bimodal MIC distribution was observed for chlorhexidine, which might point towards the acquisition of the respective resistance properties. Previous studies confirmed that biocide-like disinfectants and surfactants are effective to select for AMR [71].

In our study, the newly developed oligonucleotide microarray offered an accurate and rapid solution to detect a large set of *E. coli* VAGs. Previous studies compared the accuracy and time needed to perform a microarray-based method with conventional multiplex PCR [72], and showed that microarray-based diagnostics was less labor-intensive and, therefore, more cost-effective. In addition, the error rates occurring in the amplification process during multiplex PCR do not exist when using microarrays [73]. Therefore, in our study, microarray technology offered an accurate and rapid tool to detect a large set of VAGs in parallel.

## 5. Conclusions

In this study, we have found porcine high-risk zoonotic *E. coli* clones that are both pathogenic and multi-drug resistant. The threat that these clones can pose to public health is derived from their AMR to critically important antibiotics for humans. Therefore, our work highlights the importance of monitoring AMR and VAGs in porcine *E. coli* isolates. This can be achieved by applying reliable, fast, economical, and easy to perform technologies such as DNA-based microarray typing. Nevertheless, preventive measures in swine farms in addition to surveillance must be applied to avoid infection of the pigs with resistant and pathogenic *E. coli* strains and to avoid their spread.

## 6. Limitations of Our Study

Data on prevalence, serotypes, and pathotypes of porcine *E. coli* in Austria and other countries were scarce, which made comparisons difficult. In our study, we were not able to compare our data on the national level because resistance in swine is not monitored yet in a harmonized way in Austria.

## Figures and Tables

**Table 1:** Pheno- and genotypic characterization of *E. coli* isolated from porcine sources.

Sample Number	Phylogroup	CH-Clonotype	ESBL Phenotype	AMR Phenotype ^1^	AMR Genotype	Virulence Genes Array	Mutations QRDR ^2^ GyrA	Mutations QRDR ParC	Mutations QRDR ParE
1450	A	27-0	ESBL	AMP	*bla*_TEM-1_, *bla*_CTX-M-1_	*fimH1*, *faeG*, *astA*, *itcA*	w.t. ^4^	w.t.	w.t.
3651	A	11-23	ESBL	AMP, CAZ, SXT	*bla*_CTX-M-1_, *sul2*, *dfr1*, *dfr17*	*fimH1*, *fimH2*, *fedA*, *estIa*, *estIb*	w.t.	w.t.	w.t.
3730	B1	23-31	n.a. ^6^	AMP, SXT	n.dt. ^5^	*fimH1*, *fimH2*, *eaeA*, *ent*	w.t.	w.t.	w.t.
4245	A	11-23	n.a.	NR	n.dt.	*fimH1*, *fimH2*, *faeG*, *estIa*, *estIb*	w.t.	w.t.	w.t.
4268	A	27-0	n.a.	TET	*tet*(A)	*fimH1*, *faeG*, *astA*, *itcA*	n.d.	n.d.	n.d.
101_76	A	27-0	n.a.	AMP, PIP, SXT	*sul2*, *dfr1*	*fimH1*, *faeG*, *astA*, *itcA*, *hlyA*	w.t.	w.t.	w.t.
103_78	C	11-24	ESBL	AMP, PIP, TET, FEP	*bla*_CTX-M-1_, *tet*(A), *tet*(B)	*fimH1*, *estIa*	w.t.	w.t.	w.t.
104_79	B1	6-31	n.a.	TET	*tet(*A)	*fimH1*	n.d. ^3^	n.d.	n.d.
105_80	B1	1367-86	n.a.	AMP, TET, SXT, CIP	*tet*(A), *tet*(B), *sul2*, *dfr1*, *dfr12*	*fimH1*, *fimH2*	*gyrA S83L*, *gyrA S83A*, *gyrA D87N*	w.t.	w.t.
106_81	clade 1	11-53	n.a.	AMP, TET	*tet*(A)	*fimH1*, *fimH2*, *astA*, *aidA*	n.d.	n.d.	n.d.
107_82	A	11-23	n.a.	TET	*tet*(B)	*fimH1*, *fimH2*, *aidA*	n.d.	n.d.	n.d.
108_83	A	27-0	n.a.	NR	n.dt.	*fimH1*, *faeG*, *astA*, *itcA*	n.d.	n.d.	n.d.
109_84	B1	4-0	n.a.	AMP	*bla*_TEM-1_	*fimH1*, *fimH2*, *iucD*, *papC*	n.d.	n.d.	n.d.
17_1	A	7-54	n.a.	NR	n.dt.	*fimH1*, *fimH2*	n.d.	n.d.	n.d.
18_2	D	28-65	n.a.	AMP, PIP, TET, TOB	*tet*(B), *aadA1*	*fimH1*, *fimH2*, *fedA*, *astA*, *itcA*, *estIb*	w.t.	w.t.	w.t.
19_3	C	11-54	ESBL	AMP, PIP, TET, CTX, CHL, SXT, CIP	*bla*_TEM-1_, *bla*_CTX-M-1_, *tet*(B), *catA*, *sul2*, *dfr17*, *qnrS*	*fimH1*, *fimH2*, *iucD*, *papC*	*gyrA S83L*, *gyrA D87N*	*parC A56T*	w.t.
20_4	A	11-23	n.a.	TET, SXT	*tet*(A), *tet*(B), *sul2*, *dfr1*	*fimH1*	n.d.	n.d.	n.d.
21_5	A	29-32	n.a.	NR	n.dt.	*fimH1*, *fimH2*	n.d.	n.d.	n.d.
22_6	A	11-53	ESBL	AMP, PIP, CAZ	*bla*_TEM-1_, *bla*_CTX-M-1_	*fimH1*, *fimH2*	n.d.	n.d.	n.d.
23_7	G	45-97	n.a.	AMP, TET, SXT	*tet*(A), *sul1*, *sul*2, *dfr*17	*fimH1*, *fimH2*, *iucD*, *papC*, *pic*	w.t.	w.t.	w.t.
24_8	B2	40-22	n.a.	AMP, PIP, TET, CTX, FEP, ATM	*tet*(A)	*fimH1*, *fimH2*, *iucD*, *papC*	w.t.	w.t.	w.t.
25_9	A	11-41	n.a.	AMP, TET	n.dt.	*fimH1*	n.d.	n.d.	n.d.
27_11	B1	4-31	n.a.	AMP	*bla*_TEM-1_	*fimH1*, *fimH2*	n.d.	n.d.	n.d.
28_12	F	88-58	n.a.	AMP, TET, GEN, SXT, CIP	*tet*(B), *cml*A, *sul2*, *dfr*17	*fimH1*, *fimH2*, *astA*, *faeG*, *iucD*, *papC*	*gyrA S83L*, *gyrA D87N*	*parC E84G*	*parE I355T*
29_13	F	88-54	n.a.	TET, GEN, SXT, CIP	*tet*(B), *sul*2, *dfr*17	*fimH1*, *fimH2*, *astA*, *iucD*, *papC*	*gyrA S83L*, *gyrA D87N*	*parC E84G*	*parE I355T*
26_10	B1	19-86	AmpC	AMP, TET, CAZ	*bla*_CMY-2_, *bla*_TEM-1_	*fimH1*	n.d.	n.d.	n.d.
2945_3	C	4-54	n.a.	NR	n.dt.	*fimH1*, *astA*, *estIa*	w.t.	w.t.	w.t.
30_14	A	7-54	n.a.	AMP, TET	*bla*_TEM-1_, *tet*(A), *tet*(B)	*fimH1*, *fimH2*	n.d.	n.d.	n.d.
32_16	C	4-35	ESBL	AMP, PIP, TET	*bla*_TEM-1_, *tet*(A)	*fimH1*, *fimH2*, *iucD*, *papC*	n.d.	n.d.	n.d.
33_17	A	7-0	n.a.	TET	*tet*(A)	*fimH1*	n.d.	n.d.	n.d.
34_18	C	4-24	n.a.	AMP, PIP, CTX, FEP, GEN, CHL, CIP, ATM	*bla*_TEM-1_, *tet*(A), *tet* (B), *aadA1*, *florF*	*fimH1*, *fimH2*, *iucD*, *papC*	*gyrA S83L*, *gyrA D87N*	w.t.	w.t.
35_19	A	11-54	ESBL	AMP, TET, CAZ, SXT, CIP	*tet*(A), *sul3*, *dfr1*, *qnrS*	*fimH1*, *fimH2*, *fanA*, *estIa*	*gyrA S83L*, *gyrA D87N*	w.t.	w.t.
36_20	D	11-32	n.a.	AMP, PIP, TET	*bla*_TEM-1_, *tet*(A)	*fimH1*, *fimH2*, *iucD*, *papC*	n.d.	n.d.	n.d.
37_21	A	11-27	n.a.	NR	n.dt.	*fimH1*, *fimH2*, *astA*, *estIa*, *stxa2*	w.t.	w.t.	w.t.
38_22	B1	65-32	n.a.	AMP, TET, SXT, CIP	*bla*_TEM-1_, *tet*(B), *sul2*, *dfr17*	*fimH1*, *fimH2*, *iucD*, *papC*	*gyrA S83L*, *gyrA D87N*	w.t.	w.t.
39_23	C	11-23	n.a.	AMP, TET, CHL, SXT, CIP	*bla*_TEM-1_, *tet*(A), *cml*-A1, *sul3*, *florF*, *dfr1*, *dfr12*	*fimH1*, *fimH2*	*gyrA S83L*, *gyrA D87N*	w.t.	w.t.
40_24	B1	23-158	n.a.	TET	*tet*(B)	*fimH1*, *fimH2*, *fasA*, *estIa*	n.d.	n.d.	n.d.
40541_1	C	4-27	n.a.	NR	n.dt.	*fimH1*, *fimH2*, *iucD*, *papC*	n.d.	n.d.	n.d.
40541_2	A	4-27	n.a.	AMP, PIP, TET	*bla*_TEM-1_, *tet*(A)	*fimH1*, *faeG*, *astA*, *itcA*	w.t.	w.t.	w.t.
41_25	B1	4-57	n.a.	NR	n.dt.	*fimH1*, *fimH2*, *hlyA*	n.d.	n.d.	n.d.
42_26	A	11-54	n.a.	AMP, TET	*bla*_TEM-1_, *tet*(B)	*fimH1*, *fimH2*, *hlyA*	n.d.	n.d.	n.d.
43_27	A	11-54	n.a.	AMP, TET	*bla*_TEM-1_, *tet*(A)	*fimH1*, *fimH2*	w.t.	w.t.	w.t.
4347_1	B1	579-0	n.a.	AMP, TET, CHL, GEN, TOB	*tet*(A), *aadA1*	*fimH1*, *estIa*, *fasA*	n.d.	n.d.	n.d.
4347_2	B1	579-0	n.a.	AMP, TET	*bla*_TEM-1_, *tet*(A)	*fimH1*, *estIa*, *fasA*	n.d.	n.d.	n.d.
4347_3	B1	579-0	n.a.	TET, FOF	*bla*_TEM-1_, *tet*(A)	*fimH1*, *estIa*, *fasA*	n.d.	n.d.	n.d.
44_28	C	4-39	ESBL	AMP, PIP, TET, CAZ, SXT	*bla*_TEM-1_, *tet*(A), *sul2*, *sul3*	*fimH1*, *fimH2*, *iucD*, *papC*	n.d.	n.d.	n.d.
448_1	A	27-0	ESBL	AMP, CAZ, FEP, ATM	*bla*_TEM-1_, *bla*_CTX-M-1_	*fimH1*, *faeG*, *astA*, *itcA*	w.t.	w.t.	w.t.
448_2	A	27-0	ESBL	AMP, PIP, CTX, FEP, ATM	*bla*_TEM-1_, *bla*_CTX-M-1_	*fimH1*, *faeG*, *astA*, *itcA*	n.d.	n.d.	n.d.
45_29	A	11-0	ESBL	AMP, TET, CAZ	*bla*_TEM-1_, *bla*_CTX-M-1_, *tet*(A)	*fimH1*, *fimH2*	n.d.	n.d.	n.d.
46_30	A	11-23	n.a.	TET	*tet*(A)	*fimH1*, *fimH2*, *aidA*, *stx2e*	w.t.	w.t.	w.t.
47_31	A	11-0	n.a.	AMP, TET, GEN, TOB, CIP	*tet*(B), *aac3′-II*, *aac5-lb-cr*	*fimH1*, *astA*, *iucD*, *papC*	*gyrA S83L,* *gyrA D87N*	w.t.	w.t.
48_32	A	11-25	ESBL	AMP, PIP, CTX	*bla*_TEM-1_, *bla*_CTX-M-1_	*fimH1*, *fimH2*	n.d.	n.d.	n.d.
49_33	B1	4-86	n.a.	AMP, TET	*tet*(A), *tet*(G)	*fimH1*, *fimH2*, *iucD*, *papC*	n.d.	n.d.	n.d.
50_34	B1	23-158	n.a.	TET	*tet*(A)	*fimH1*, *fimH2*, *pic*	n.d.	n.d.	n.d.
51_15	B1	19-32	n.a.	AMP, TET	*tet*(B)	*fimH1*	w.t.	w.t.	w.t.
566_1	D	28-41	n.a.	NR	n.dt.	*fimH1*, *fimH2*, *fedA*, *estIa*, *estIb*, *aidA*, *hlyA*, *stxa2*, *stx2e*	w.t.	w.t.	w.t.
566_2	D	28-41	n.a.	NR	n.dt.	*fimH1*, *fimH2*, *fedA*, *estIa*, *aidA*, *hlyA*, *stxa2*, *stx2e*	n.d.	n.d.	n.d.
566_3	D	28-41	n.a.	NR	n.dt.	*fimH1*, *fimH2*, *fedA*, *estIa*, *aidA*, *hlyA*, *stxa2*, *stx2e*	n.d.	n.d.	n.d.
60_35	A	11-54	n.a.	AMP, PIP, TET, CTX, CHL, SXT, CIP	*bla*_TEM-1_, *tet*(A), *sul1*, *sul2*, *sul3*, *dfr1*, *dfr12*, *dfr17*, *catA*, *cmlA1*	*fimH1*, *fimH2*	*gyrA S83L,* *gyrA D87N*	w.t.	w.t.
61_36	B1	19-38	n.a.	AMP, TET, GEN, SXT, CIP	*bla*_TEM-1_, *tet*(A), *tet*(B), *sul1*, *sul*2, *dfr*17, *aadA1*, *aadA5*, *qnrS*	*fimH1*, *fimH2*, *iucD*, *papC*	*gyrA S83L*, *gyrA D87N*	w.t.	w.t.
62_37	A	7-54	n.a.	AMP, TET	*bla*_TEM-1_, *tet*(A), *tet*(B)	*fimH1*, *fimH2*	*n.d.*	n.d.	n.d.
63_38	B1	7-31	n.a.	AMP, PIP, TET, CTX, CIP	*bla*_TEM-1_, *tet*(A), *qnr*S	*fimH1*, *fimH2*, *astA*	*gyrA S83L,* *gyrA D87N*	w.t.	w.t.
630_2	A	27-0	n.a.	NR	n.dt.	*fimH1*, *fimH2*, *faeG*, *astA*, *itcA*	n.d.	n.d.	n.d.
64_39	B2	52-5	ESBL	AMP, PIP, TET, CTX	*bla*_TEM-1_, *tet*(A)	*fimH1*, *fimH2*, *astA*, *papC*, *iucD*, *cnf1*	n.d.	n.d.	n.d.
65_40	A	11-0	ESBL	AMP, TET, SXT, CTX?, CAZ	*bla*_TEM-1_, *bla*_CTX-M-1_, *tet*(A), *tet*(B), *sul*2	*fimH1*	n.d.	n.d.	n.d.
66_41	B1	41-54	ESBL	AMP, PIP, TET, CTX, FEP, SXT, ATM	*bla*_TEM-1_, *bla*_CTX-M-1_, *tet*(A), *tet*(B), *sul2*, *dfr1*, *dfr12*	*fimH1*, *fimH2*	w.t.	w.t.	w.t.
67_42	A	27-0	n.a.	AMP	n.dt.	*fimH1*, *astA*, *faeG*, *itcA*, *hlyA*	w.t.	w.t.	w.t.
68_43	A	27-0	n.a.	AMP, PIP	n.dt.	*fimH1*, *astA*, *faeG*, *itcA*, *hlyA*	w.t.	w.t.	w.t.
69_44	A	41-38	n.a.	TET	*tet*(B)	*fimH1*, *fimH2*, *iucD*, *papC*	w.t.	w.t.	w.t.
70_45	B1	579-0	n.a.	AMP, CHL, SXT	*sul2*, *dfr1*, *catA*	*fimH1*, *estIa*, *fasA*	w.t.	w.t.	w.t.
71_46	A	11-54	n.a.	AMP, PIP	*bla*_TEM-1_	*fimH1*	n.d.	n.d.	n.d.
72_47	A	11-45	n.a.	NR	n.dt.	*fimH1*, *fimH2*, *astA*, *aidA*	n.d.	n.d.	n.d.
73_48	A	11-45	n.a.	CHL, SXT?	*cmlA1*	*fimH1*, *fimH2*, *astA*, *aidA*	w.t.	w.t.	w.t.
74_49	A	11-23	n.a.	AMP, TET	*bla*_TEM-1_, *tet*(A)	*fimH1*, *fimH2*	n.d.	n.d.	n.d.
75_50	A	11-24	n.a.	AMP, TET	*bla*_TEM-1_, *tet*(A), *tet*(B)	*fimH1*, *fimH2*	n.d.	n.d.	n.d.
76_51	A	27-0	n.a.	NR	n.dt.	*fimH1*, *faeG*, *astA*, *itcA*	w.t.	w.t.	w.t.
77_52	E	7-31	n.a.	NR	n.dt.	*fimH1*, *fimH2*	n.d.	n.d.	n.d.
78_53	A	11-23	n.a.	TET	*tet*(A)	*fimH1*, *fimH2*	n.d.	n.d.	n.d.
79_54	A	11-24	n.a.	TET	*tet*(A)	*fimH1*, *fimH2*	n.d.	n.d.	n.d.
80_55	A	11-54	n.a.	TET, CHL	*tet*(A), *cmlA1*	*fimH1*, *fimH2*	n.d.	n.d.	n.d.
81_56	A	4-24	n.a.	NR	n.dt.	*fimH1*, *fimH2*	n.d.	n.d.	n.d.
82_57	B1	6-289	n.a.	NR	n.dt.	*fimH1*, *fimH2*, *astA*, *fedA*, *aidA*, *stx2e*	w.t.	w.t.	w.t.
83_58	B2	11-25	n.a.	AMP, PIP, FOF	*bla*_TEM-1_, *fosB*	*fimH1*, *cnf1*	n.d.	n.d.	n.d.
84_59	B1	23-158	ESBL	AMP, CTX, FEP	*bla*_TEM-1_ *bla*_CTX-M-1_	*fimH1*, *fimH2*	n.d.	n.d.	n.d.
85_60	A	4-27	ESBL	AMP, PIP, TET, CTX, CAZ, FEP	*bla*_TEM-1_, *tet*(B)	*fimH1*, *iucD*, *papC*	n.d.	n.d.	n.d.
86_61	B1	41-0	n.a.	NR	n.dt.	*fimH1*, *fimH2*, *iucD*, *papC*	n.d.	n.d.	n.d.
87_62	C	11-35	ESBL	AMP, PIP	*bla*_TEM-1_	*fimH1*, *iucD*, *papC*	n.d.	n.d.	n.d.
88_63	A	11-25	n.a.	AMP, CTX	*bla*_TEM-1_, *bla*_CTX-M-1_	*fimH1*, *fimH2*	n.d.	n.d.	n.d.
89_64	A	27-0	n.a.	TET, SXT, CIP	*tet*(A), *sul1*, *sul2*, *dfr1*	*fimH*, *astA*, *iucD*, *papC*	*gyrA S83L,* *gyrA D87N*	w.t.	w.t.
90_65	A	27-0	n.a.	TET, GEN, TOB,	*tet*(A), *aadA1*, *aadA*2, *aadA5*	*fimH1*, *faeG*, *astA*, *itcA*	n.d.	n.d.	n.d.
91_66	A	11-25	n.a.	NR		*fimH1*, *fimH2*	n.d.	n.d.	n.d.
92_67	A	11-398	ESBL	AMP, TET	*bla*_TEM-1_, *tet*(B)	*fimH1*, *fimH2*	n.d.	n.d.	n.d.
93_68	B1	4-32	ESBL	AMP, TET, CHL, CIP	*bla*_TEM-1_, *tet*(C), *catA*, *florF*, *cmlA*	*fimH1*, *fimH2*, *iucD*, *papC*	*gyrA S83L,* *gyrA D87N*	w.t.	w.t.
94_69	B1	41_86	n.a.	AMP, TET, SXT	*bla*_TEM-1_, *tet*(A), *sul1*, *sul2*, *aadA1*	*fimH1*, *fimH2*	w.t.	w.t.	w.t.
95_70	A	11-34	ESBL	AMP, PIP, TET	*bla*_TEM-1_, *bla*_CTX-M-1_, *tet*(A), *tet*(B)	*fimH1*, *fimH2*	n.d.	n.d.	n.d.
96_71	B1	4-27	n.a.	NR	n.dt.	*fimH1*, *iucD*, *papC*	n.d.	n.d.	n.d.
97_72	A	27-54	n.a.	TET	*tet*(A)	*fimH1*, *fimH2*	n.d.	n.d.	n.d.
98_73	A	7-54	n.a.	NR	n.dt.	*fimH1*, *fimH2*, *astA*, *aidA*, *bfpB*	w.t.	w.t.	w.t.
99_74	B2	40-22	n.a.	AMP, PIP, TET, SXT	*tet*(A), *dfr1*, *dfr17*	*fimH1*, *fimH2*, *papC*, *iucD*, *cnf1*	w.t.	w.t.	w.t.
3835_2	B1	4-440	n.a.	AMP, PIP, TET, CHL, SXT, COL	*mcr1*	*fimH1*, *fimH2*, *astA*, *eaeA*, *ent*, *escV*, *hlyA*	w.t.	w.t.	w.t.
3835_3	A	11-54	n.a.	AMP, PIP, TET, CHL, SXT, COL	*mcr1*	*fimH1*, *fimH2*	w.t.	w.t.	w.t.
3835_4	A	11-54	n.a.	AMP, PIP, TET, CHL, SXT, COL	*mcr1*	*fimH1*, *fimH2*	w.t.	w.t.	w.t.

^1^ Abbreviations: AMC, amoxicillin/clavulanate; CAZ, ceftazidime; CHL, chloramphenicol; CIP, ciprofloxacin; CFZ, cefazolin; CTX, cefotaxime; FOF, fosfomycin; GEN, gentamicin; PIP, piperacillin; SXT, trimethoprim/sulfamethoxazole; TET, tetracycline; TOB, tobramycin; COL, colistin; NR, not resistant. ^2^ QRDR: quinolone-resistance-determining region. ^3^ n.d., not done. ^4^ w.t., wild type. ^5^ n.dt., none detected using the primer-set of this study. ^6^ n.a., not applicable.

**Table 2:** Characterization of whole-genome-sequenced porcine *E. coli*.

Isolate	Phylogroup	CH-Clonotype	Serotype ^1^	Sequence-Type	ESBL ^6^	AMR Phenotype ^2^	WGS AMR Genes	WGS VAG	QRDR ^4^ *GyrA* ^3^	QRDR ^4^ *ParC* ^3^	QRDR ^4^ *ParE* ^3^
1450	A	27-0	O_NT_:H10	clustered 100	ESBL	AMP	*bla*_TEM-1B_ *, *bla*_CTX-M-1_ *, *mdfA*, *mphA*	faeG *, astA *, *capU*, ***cba****^5^*, ***cia***, cma *, *gad*, *iha*, ItcA *, stb *, *terC*, ***traT***	w.t.	w.t.	w.t.
3651	A	11-23	O_NT_:H32	10	ESBL	AMP, CAZ, SXT	***bla*_CTX-M-1_**, ***sul2***, ***dfrA17***, ***aadA5***, *mdfA*	***cib***, ***fedA***, ***fedF***, ***gad***, *iss*, *ompT*, *sta1*, *stb*, *terC*, ***traT***	w.t.	w.t.	w.t.
3730	B1	23-31	O_NT_:H21	56	n.a. ^7^	AMP, SXT	***bla*_TEM-1B_**, sul1 *, sul2 *, dfrA1 *, aadA1 *, aph(3″)-Ib *, aph(6)-Id *, *mdfA*, ***mphB***	***cma***, ***cvaC***, *gad*, ***hlyF***, ***iroN***, ***iss***, *IpfA*, *ompT*, *sitA*, *terC*, ***traT***	w.t.	w.t.	w.t.
4245	A	11-23	O_NT_:H26	1112	n.a.	NR	sul1 *, aadA1 *	***faeG***, ***cea***, ***cib***, *gad*, sepA*, sta1 *, *stb*, *terC*, ***traT***	w.t.	w.t.	w.t.
101_76	A	27-0	O_NT_:H10	clustered 100	n.a.	AMP, PIP, SXT	*bla*_TEM-1C_ *, *mdfA*, sul2 *, dfrA1 *, *qnrD1* *, aph(3″)-Ib *, aph(6)-Id *	***faeG***, ***astA*,** *capU*, *gad*, *iha*, *stb*, *terC*, *traT*	w.t.	w.t.	w.t.
103_78	C	11-24	O_NT_:H12	10	ESBL	AMP, PIP, TET, FEP	***bla*_CTX-M-1_**, *tet*(B) *, sul1 *, ***mdfA***, *mphA*, aadA1 *,	***cia***, *hra*, *iha*, *iroN*, *ompT*, ***papC***, *terC*, ***traT***	w.t.	w.t.	w.t.
18_2	D	28-65	O108:H4	42	n.a.	AMP, PIP, TET, TOB	*bla*_TEM-1B_ *, *tet*(B) *, aac(3)-IV *, *aadA1*, *aph(3″)-Ib* *, aph(6)-Id *, aph(4)-Ia *, *mdfA*	*air*, *astA*, *chuA*, ***fedA***, ***fedF***, *hra*, *iha*, *iss*, *IpfA*, *ItcA*, *neuC*, *ompT*, *stb*, *terC*, ***traT***	w.t.	w.t.	w.t.
19_3	C	11-54	O_NT_:H10	744	ESBL	AMP, PIP, TET, CTX, CHL, SXT, CIP	*tet*(B) *, ***sul1***, *sul2* *,***dfrA17***, aph(3′)-Ia *, *aph*(3″)-Ib *, *aph(6)-Id* *, ***aadA5***, *cat*A1 *, *mdfA*	***cba***, ***cia***, cma *, *cvaC*, etsC *, *gad*, ***hlyF***, *iroN*, ***iss***, ***iucC***, ***iutA***, *mchF*, ompT *, *sitA*, *terC*, ***traT***, tsh *	*gyrA S83L*, *gyrA D87N*	*par*C A56T	w.t.
23_7	G	45-97	O_NT_:H4	117	n.a.	AMP, TET, SXT	***bl*a_TEM-1B_**, ***sul1***, *sul*2 *, *tet*(A) *, ***dfrA17***, *mdfA*, ***mphA***, aph(3″)-Ib *, aph(6)-Id *, ***aadA5***	*cea*, *chuA*, *fyuA*, *gad*, *hlyE*, ***hlyF***, *ireA*, *iroN*, *irp2*, *iss*, ***iucC***, ***iutA***, *katP*, *IpfA*, ***ompT***, *pic*, ***sitA***, *terC*, ***traT***, *vat*	w.t.	w.t.	w.t.
24_8	B2	40-22	025:H4	131	n.a.	AMP, PIP, TET, CTX, FEP, ATM	***bla*_TEM-1C_**, *bla*_CTX-M-1_ *, *tet*(A) *, ***aph(3′)-Ia***, *mphA* *, *mdfA*, qnrS1 *	*chuA*, ***cia***, cvaC *, *etsC*, *fyuA*, *gad*, ***hlyF***, *hra*, *ibeA*, *iroN*, *irp2*, *iss*, ***iucC***, ***iutA***, *kpsE*, *kpsMII*, mchF *, ***ompT***, *papA-F48*, *papC*, *sitA*, *terC*, ***traT***, *usp*, *yfcV*	w.t.	w.t.	w.t.
28_12	F	88-58	O_NT_:H34	354	n.a.	AMP, TET, GEN, SXT, CIP	***bla*_TEM-1B_**, *sul*2 *, *tet*(B) *, *dfrA17* *, *aph(3″)-Ib* *, *aph(6)-Id* *, *aac(3)-IId*, aph(3′)-Ia *, *mdfA*	*air*, *astA*, *chuA*, *eiIA*, *gad*, *hra*, *ibeA*, ***iucC***, ***iutA***, *kpsE*, *kpsMII_K5*, *IpfA*, ***sitA***, *terC*, *usp*, *yfcV*	*gyrA S83L*, *gyrA D87N*	*parC E84G*	*parE I355T*
29_13	F	88-54	O_NT_:H34	354	n.a.	TET, GEN, SXT, CIP	*tet*(B) *, *sul*2 *, ***dfr*A17**, aph(3″)-Ib *, *aac(3)-IId*, aph(6)-Id *, *mdfA*	*air*, *astA*, *chuA*, *eiIA*, *gad*, *hra*, *ibeA*, ***iucC***, ***iutA***, *kpsE*, ***kpsMII_K5***, *IpfA*, ***sitA***, *terC*, *usp*, *yfcV*	*gyrA S83L*, *gyrA D87N*	*parC E84G*	*parE I355T*
2945_3	C	4-54	O8:H17	23	n.a.	NR	*mdfA*	*asta*, cia *, *fanA*, *fyuA*, *gad*, *irp2*, *iss*, *IpfA*, ***mcbA***, *ompT*, ***sepA***, *terC*, ***traT***	w.t.	w.t.	w.t.
35_19	A	11-54	O_NT_:H9	10	ESBL	AMP, TET, CAZ, SXT, CIP	*bla*_TEM-52B_ *, ***tet*(B)**, sul1 *, dfrA1 *,***aph(3″)-Ib***, *aadA1*, ***aph(6)-Id***, *mdfA*	***cia***, ***cib***, ***fanA***, *gad*, *iss*, *terC*, traT *	*gyrA S83L*, *gyrA D87N*	w.t.	w.t.
37_21	A	11-27	O_NT_:H16	neuer ST	n.a.	NR	*mdfA*	***astA***, *gad*, *iha*, *iss*, *IpfA*, ***sepA***, ***sta1***, ***stb***, *stx2A*, *stx2B*, *terC*, *traT*, *stx2*	w.t.	w.t.	w.t.
40541_2	A	4-27	n.t.	100	n.a.	AMP, PIP, TET	***bla*_TEM-1B_**, *tet*(A) *, *sul*2 *, dfrA14 *, *mdfA*, aph(3″)-Ib *, *aph(6)-Id* *	faeG *, astA *, *capU*, ***cib***, *gad*, *iha*, ItcA *, stb *, *terC*, *traT*	w.t.	w.t.	w.t.
4347_1	B1	579_0	O64:H-	6404	n.a.	AMP, TET, CHL, GEN, TOB	*bla*_TEM-1B_ *, ***tet*(A)**, ***sul1***, *qnrS1*, *aph(3″)-Ib*, *aph(6)-Id*, aph(4)-Ia *, aac(3)-IV *, ***aadA1***, *mdfA*, catA1 *	***cba***, ***cea***, *cma*, *fasA*, *gad*, *iss*, *IpfA*, *ompT*, *terC*	w.t.	w.t.	w.t.
448_1	A	27-0	O_NT_:H10	clustered 100	ESBL	AMP, CAZ, FEP, ATM	***bla*_CTX-M-1_** *, *bla*_TEM-1B_, mphA *, *mdfA*	faeG *, *astA*, *capU*, ***cba***, ***cia***, *cma*, *gad*, *iha*, *ItcA*, *stb*, *terC*, ***traT***	w.t.	w.t.	w.t.
46_30	A	11-23	O142:H27	neu icd	n.a.	TET	*tet*(A), *mdfA*	*stx2*, ***sepA***, *stx2A*, *stx2B*, *terC*, ***traT***	w.t.	w.t.	w.t.
51_15	B1	19-32	O_NT_ H49	1079	n.a.	AMP, TET	*bla*_TEM-1B_ *, *tet*(B) *, *mdfA*, *aph(3″)-Ib*, *aph(6)-Id*	*gad*, *IpfA*, *terC*	w.t.	w.t.	w.t.
566_1	D	28-41	O138:H14	760	n.a.	NR	*mdfA*	*stx2*, *chuA*, ***fedA***, ***fedF***, *gad*, *hra*, *iha*, *iss*, *ompT*, *sta1*, *stb*, *stx2A*, *stx2B*, *terC*, ***traT***	w.t.	w.t.	w.t.
66_41	B1	41-54	O88:H21	101	ESBL	AMP, PIP, TET, CTX, FEP, SXT, ATM	*bla*_TEM-1B_ *, **bla_CTX-M-1_**, *tet*(B) *, *dfrA1* *, *aadA1* *, qnrS1 *, *mdfA*, ***mphA***	*gad*, *hra*, *iss*, *IpfA*, *ompT*, *terC*	w.t.	w.t.	w.t.
67_42	A	27-0	O_NT_:H10	clustered 100	n.a.	AMP	***bla*_TEM-1B_**, *mdfA*	***faeG***, ***astA***, ***capU***, ***cba***, ***cia***, *cma*, *gad*, *iha*, ***ItcA***, *stb*, *terC*, ***traT***	w.t.	w.t.	w.t.
68_43	A	27-0	O_NT_:H10	clustered 100	n.a.	AMP, PIP	***bla*_TEM-1B_**, *mdfA*	faeG *, *astA*, *capU*, ***cba***, ***cia***, *cma*, *gad*, *iha*, *ItcA*, *stb*, terC traT *	w.t.	w.t.	w.t.
69_44	A	41-38	O_NT_:H21	101	n.a.	TET	*tet*(B) *, *mdfA*, *aph(3″)-Ib*, *aph(6)-Id*	***cia***, cvaC *, *etsC*, *gad*, ***hlyF***, *iroN*, *iss*, ***iucC***, ***iutA***, *IpfA*, *ompT*, ***sitA***, *terC*, ***traT***	w.t.	w.t.	w.t.
70_45	B1	579-0	n.t.	6404	n.a.	AMP, CHL, SXT	***bla*_TEM-1B_**, ***sul1***, sul2 *, dfrA1 *, ***aadA1***, aph(3″)-Ib *, *aph(6)-Id* *, *mdfA*, ***catA1***	***cba***, ***cea***, ***cia***, *cma*, *fasA*, *gad*, *iss*, *IpfA*, *ompT*, *terC*, *traT*	w.t.	w.t.	w.t.
73_48	A	11-45	O_NT_:H6	10	n.a.	CHL, SXT	***cmlA1***,***sul3***, ***dfrA12***, ***aadA2***, ***aadA1***, *mdfA*	***astA***, *gad*, *stb*, *terC*	w.t.	w.t.	w.t.
76_51	A	27-0	O_NT_:H10	100	n.a.	NR	*sul*2 *, *mdfA*, aph(6)-Id *, aph(3″)-Ib *	faeG *, ***astA***, *capU*, *cba*, *cma*, *gad*, *iha*, *terC*, ***traT***	w.t.	w.t.	w.t.
82_57	B1	6-289	O121:H10	641	n.a.	NR	*mdfA*	***stx2***, ***astA***, ***fedA***, ***fedF***, *gad*, *IpfA*, ***sepA***, ***stx2A***, ***stx2B***, *terC*, ***traT***	w.t.	w.t.	w.t.
94_69	B1	41-86	O82:H8	6365	n.a.	AMP, TET, SXT	*mdfA*, ***sul1***, ***tet*(C)**, ***aadA1***	***cea***, *cnf1*, *cvaC*, etsC *, *gad*, hlyF *, *hra*, *iroN*, *iss*, ***iucC***, ***iutA***, *IpfA*, *mchF*, ompT *, *papA-F1651A*, *papC*, ***sitA***, *terC*, ***traT***, *tsh*	w.t.	w.t.	w.t.
98_73	A	7-54	O_NT_:H10	neu icd	n.a.	NR	*mdfA*	***astA***, *fyuA*, *irp2*, *papC*, ***stb***, *terC*, ***traT***	w.t.	w.t.	w.t.
99_74	B2	40-22	O25:H4	131	n.a.	AMP, PIP, TET, SXT	***bla*_TEM-1C_**, ***aadA1***, *mdfA*, *tet*(A) *, ***dfrA1***, ***sul3***	***cea***, *chuA*, ***cia***, *cnf1*, *cvaC*, *etsC*, *fyuA*, *gad*, ***hlyF***, *hra*, *ibeA*, *iroN*, *irp2*, *iss*, ***iucC***, ***iutA***, *kpsE*, *kpsMII_K5*, *mchF*, ***ompT***, *papA_F14*, *papC*, ***sitA***, *terC*, ***traT***, *usp*, *yfcV*	w.t.	w.t.	w.t.
3835_2	B1	4-440	O26:H11	88	n.a.	AMP, PIP, TET, CHL, SXT, COL	aadA1 *, aadA2 *, cmlA1 *, mcr-1.1 *, *tet*(A) *, ***tet*(M)**, mefB *, *mdfA*, dfrA12 *, **bla_TEM-1B_**, sul3 *	*astA*, ***cif***, *eaE*, *efa1*, ***ehxA***, ***espP***, *espA*, *espB*, *espF*, *espJ*, *espP*, *fyuA*, *gad*, *iha*, *irp2*, *iss*, ***katP***, *IpfA*, *nleA*, *nleB*, *ompT*, *terC*, *tir*, traT *	w.t.	w.t.	w.t.
3835_3	A	11-54	O2:H2	10	n.a.	AMP, PIP, TET, CHL, SXT, COL	*tet*(A) *, ***sul*3**, ***aph(3″)-Ib***, aadA2 *, ***aph(6)-Id***, *mdf*(A), dfrA12 *, cmlA1 *, ***mcr-1.1***, ***bla*_TEM-1D_**	***cea***, *cvaC*, *gad*, *hra*, *iha*, *iss*, ***katP***, *mchF*, *terC*, traT *	w.t.	w.t.	w.t.
3835_4	A	11-54	O2:H2	10	n.a.	AMP, PIP, TET, CHL, SXT, COL	*tet*(A) *, sul3 *, ***aph(3″)-Ib***, ***aadA2***, ***aph(6)-Id***, *mdf*(A),***dfrA12***, ***cmlA1***, ***mcr-1.1****,* ***bla*_TEM-1D_**	***cea***, *cvaC*, *gad*, *hra*, *iha*, *iss*, ***katP***, *mchF*, *terC*, ***traT***	w.t.	w.t.	w.t.

^1^ n.t., not typeable. ^2^ Abbreviations: AMC, amoxicillin/clavulanate; CAZ, ceftazidime; CHL, chloramphenicol; CIP, ciprofloxacin; CTX, cefotaxime; FOF, fosfomycin; GEN, gentamicin; PIP, piperacillin; SXT, trimethoprim/sulfamethoxazole; TET, tetracycline; TOB, tobramycin; COL, colistin. ^3^ w.t., wild type. ^4^ QRDR: quinolone-resistance-determining region; w.t., wild type. ^5^ bold letters: plasmid predicted by mlplasmids. ^6^ ESBL: Extended-spectrum β-lactamase. ^7^ n.a., not applicable. * potentially plasmid-encoded as deduced from BLASTn analyses.

## Data Availability

All data are contained within the article or Supplementary Materials.

## Supplementary Materials

The following are available online at https://www.mdpi.com/article/10.3390/microorganisms9081676/s1, Tables S1 and S2: Biocide susceptibility, Table S3: Plasmid presence_virulence genes_sorted, Table S4: Plasmid probability prediction—AMR genes, Table S5: Plasmid probability prediction_virulence genes, Table S6: Overview plasmid presence AMR genes. Figure S1: Splitstree.
