# Supplementary material for: The Pheno- and Genotypic Characterization of Porcine Escherichia coli Isolates
Source: Microorganisms. 2021 Aug 6;9(8):1676. doi: 10.3390/microorganisms9081676 (PMC8400056; doi:10.3390/microorganisms9081676)
Supplement: Supplementary file 1 [file microorganisms-09-01676-s001.zip › Supplementary Material_final/Table_S1_and_S2_biocide_susceptibility.pdf]

Table S1: Results of the biocides susceptibility testing of *E. coli* isolated from pigs

Minimal inhibitory concentration (MIC)											
**RSMB** [1]	**BENZALKONIUM CHLORIDE (BAC)**										
≥0.004%	0.000015%	0.00003%	0.00006%	0.000125%	0.00025%	0.0005%	0.001%	0.002%	0.004%	0.008%	0.016%
No of isolates						2	59	43			
**RSMB**	**CHLORHEXIDINE (CHX)**										
≥0.00025%	0.000015%	0.00003%	0.00006%	0.000125%	0.00025%	0.0005%	0.001%	0.002%			
No of isolates		12	28	13	23	18	9	1			
**RSMB**	**GLUTARDIALDEHYDE (GLU)**										
≥0.5%	0.0075%	0.015%	0.03%	0.06%	0.125%	0.25%	0.5%	1%			
No of isolates			4	49	28	21	2				
**RSMB**	**ISOPROPANOL (ISO)**										
≥10%	<1%	1%	2%	4%	6%	8%	10%	12%	14%		
No of isolates	1		6	26	54	16	1				

Abbreviations: [1] Reduced susceptibility MIC breakpoint based on the established protocol of Schug et al. [21]

Table S2: Established MIC values of investigated biocides on reference strains

	**BAC**[5] **%**		**CHX**[6] **(%)**		**ISO**[7] **(%)**		**GLU**[8] **(%)**	
	"Acceptable ranges – 24h"* [22]	Established MIC	"Acceptable ranges – 24h" [22]	Established MIC	"Acceptable ranges – 24h" [22]	Established MIC	"Acceptable ranges – 24h" [22]	Established MIC
S.[1] ***aureus*** **ATCC**[9] **6538**	0.00006-0.00025	0.0003	0.00003-0.000125	0.00003	4-10	8	0.03-0.25	0.06
E.[2] ***hirae*** **ATCC 10541**	0.000125-0.0005	0.0006	0.00003-0.00025	0.00006	4-10	8	0.06-0.25	0.125
E.[3] ***coli*** **ATCC 10536**	0.0005-0.002	0.0001	0.000016-0.000125	0.00006	2-8	4	0.03-0.25	0.125
P.[4] ***aeruginosa*** **ATCC 15442**	0.002-0.0016	0.004	0.001-0.004	0.00025	1-6	4	0.03-0.125	0.125

Abbreviations: [1] *S.*, *Staphylococcus*, [2] *E.*, *Enterococcus*, [3] *E.*, *Escherichia*, [4] *P.*, *Pseudomonas*, [5] BAC, Benzalkonium Chloride, [6] CHX, Chlorhexidine, [7] ISO, Isopropanol, [8] GLU, Glutardialdehyde, [9] ATCC, American Type Culture Collection

* based on the most common values +/- one dilution steps obtained for the different subgroups after incubation of 24 h.
