# Supplementary figures and images for: The Pheno- and Genotypic Characterization of Porcine Escherichia coli Isolates
Source: Microorganisms. 2021 Aug 6;9(8):1676. doi: 10.3390/microorganisms9081676 (PMC8400056; doi:10.3390/microorganisms9081676)

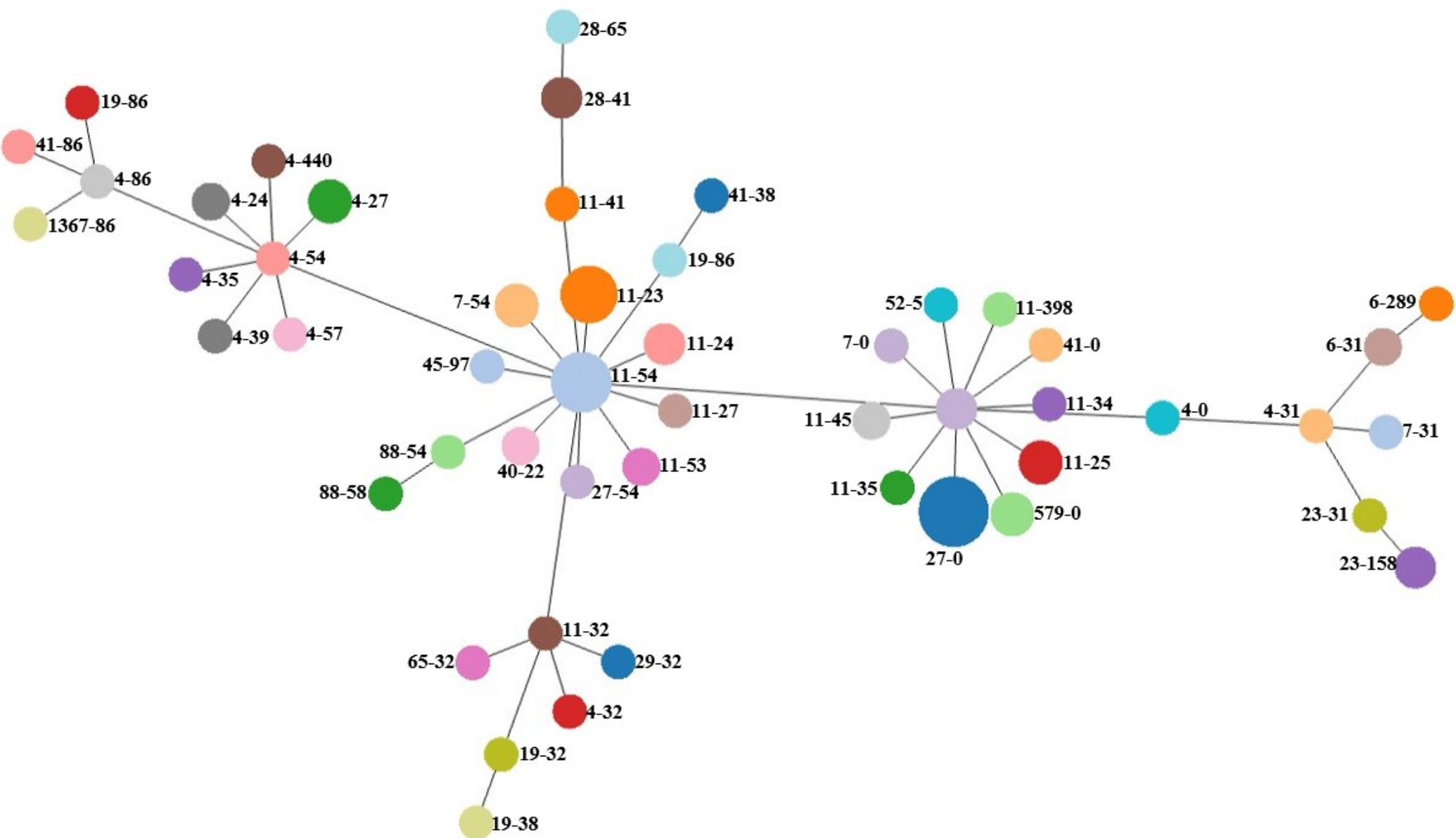

Supplement: Supplementary file 1 [file microorganisms-09-01676-s001.zip › Supplementary Material_final/CH Spanningtree.pdf]

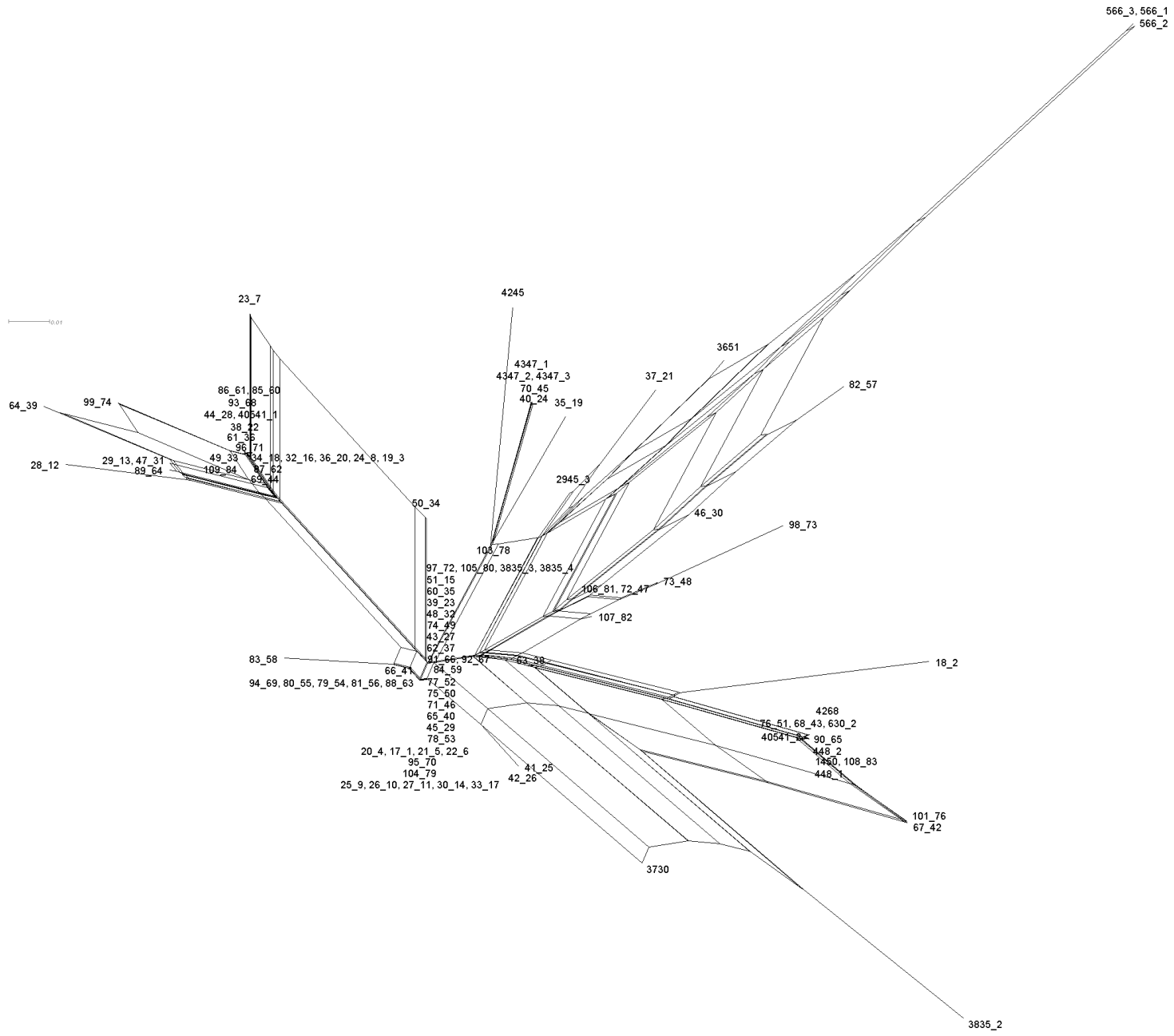

Supplement: Supplementary file 1 [file microorganisms-09-01676-s001.zip › Supplementary Material_final/Figure S1_Splitstree.pdf]
